# Understanding Experiences of Telehealth in Palliative Care: Photo Interview Study

**DOI:** 10.2196/53913

**Published:** 2025-02-11

**Authors:** Mahima Kalla, Teresa O'Brien, Olivia Metcalf, Rashina Hoda, Xiao Chen, Andy Li, Catriona Parker, Michael Edward Franco, Sam Georgy, Kit Huckvale, Christopher Bain, Peter Poon

**Affiliations:** 1 Centre for Digital Transformation of Health, Faculty of Medicine, Dentistry and Health Sciences, University of Melbourne, Melbourne Connect, 700 Swanston St, Carlton, VIC 3053, Australia, 61 0390355553; 2 Faculty of Information Technology, Monash University, Melbourne, Australia; 3 School of Information and Physical Sciences, College of Engineering, Science and Environment, University of Newcastle, Callaghan, Australia; 4 Transfusion Research Unit, Faculty of Medicine, Nursing & Health Sciences, Monash University, Clayton, Australia; 5 Faculty of Medicine, Nursing and Health Sciences, Monash University, Clayton, Australia; 6 St Vincent's Health, Melbourne, Australia; 7 Healthdirect Australia, Sydney, Australia; 8 Alliance for Digital Health At Monash, Faculty of Information Technology, Monash University, Clayton, Australia; 9 Supportive and Palliative Care Unit, Monash Health, Melbourne, Australia; 10 Department of Medicine, School of Clinical Sciences at Monash Health, Faculty of Medicine,Nursing & Health Sciences, Monash University, Melbourne, Australia

**Keywords:** consultation summary, digital scribe, qualitative research, telehealth, digital health, photo-elicitation, palliative care, photo interview, qualitative research, photographs, intertextual analysis

## Abstract

**Background:**

It is widely accepted that the COVID-19 pandemic has accelerated the era of online health care delivery, including within community palliative care. This study was part of a larger project involving a collaboration between universities, health care services, government agencies, and software developers that sought to enhance an existing telehealth (video call) platform with additional features to improve both patient and health care professional (HCP) experience in a palliative care context.

**Objective:**

The aim of this study was to understand palliative care patients’ and HCPs’ experiences of telehealth delivery in a palliative care context in Victoria, Australia. For the purposes of this study, telehealth included consultations by both video and telephone calls. By better understanding users’ experiences and perceptions of telehealth, we hoped to determine users’ preferences for new telehealth enhancement features.

**Methods:**

A total of 6 health care professionals and 6 patients were recruited from a major tertiary hospital network’s palliative care unit in Victoria, Australia. Participants were asked to generate 3‐5 photographs depicting their telehealth experiences. These photographs were used as visual aids to prompt discussion during subsequent one-on-one interviews. Intertextual analysis was conducted to identify key themes.

**Results:**

A total of 3 overarching themes emerged: comfort (or lack thereof) afforded by telehealth, connection considerations in telehealth, and care quality impacts of telehealth. Patients (n=6) described telehealth as supporting their physical and psychological comfort and maintaining connection with HCPs, yet there were specific situations where it failed to meet their needs or impacted care quality and delayed treatment. HCPs (n=6) recognized the benefit of telehealth for patients but reported several limitations of telehealth, in particular due to lack of physical examination opportunities. Participants indicated that 2 types of connection were imperative for effective telehealth delivery: technical connection (eg, good internet connectivity or clear phone line) and interpersonal connection (ie, good rapport and therapeutic alliance between the HCPs and patients). Often technical connection issues impeded the development of interpersonal connection between the HCPs and patients in telehealth.

**Conclusions:**

The findings presented in this study combined with other co-design activities, which are outside the scope of this paper, indicated the potential value of a telehealth enhancement feature that generates patient-facing clinical consultation summaries. Our team has developed a video telehealth enhancement feature (or “add-on”), which will enable clinicians to distill key actionable advice and self-management guidance discussed during teleconsultations for a take-home summary document for patients. The add-on’s prototype has also been subjected to an initial simulation study, which will be reported in a future publication.

## Introduction

In Australia, chronic illness continues to be a major challenge in health care [1] with more than three-quarters (78.6%) of Australians impacted by at least one chronic condition [2]. Many of these chronic conditions are life-limiting [3], and thus patients may benefit from palliative care [4]. The 2018 National Palliative Care Strategy recommends the approach of using palliative care as a “means to improve the quality of life of patients and their families affected with life-threatening illnesses through the prevention and relief of suffering by means of early identification, assessment, and treatment of pain and other problems” [5]. Studies have shown that palliative care improves quality of life by improving independence, providing symptom relief and pain control, and supporting the physical, emotional, and spiritual needs of patients, their carers, and loved ones [6-9]. Furthermore, palliative care is linked with a significant decrease in health care use and prolongment of survival [10].

Specialist palliative care in Australia is delivered using different models of care depending on the geographic location and available resources [11]. Many of these models of care were transformed during the COVID-19 pandemic to promote physical distancing [12,13]. One such approach for the delivery of specialist palliative care in Australia was a move to telehealth to limit hospital attendance and in-home visits [12]. The implementation of telehealth in palliative care in Australia demonstrated promise beyond the pandemic, with the potential to improve communication, facilitating more equitable access to palliative care (especially for those in rural and regional areas), avoiding lengthy travel to hospitals or clinics (particularly when patients are very unwell), and potentially assisting with timely symptom relief [14]. However, for continued sustainability and success of telehealth models, telehealth needs to adapt and evolve based on feedback from patients and health care professionals (HCPs) [15].

The importance of user involvement in the implementation of palliative care services has been recognized and prioritized by researchers to improve productivity, quality, and relevance of care [16]. The aim of this study was to understand palliative care patients’ and HCPs’ experiences of telehealth delivery in a palliative care context in Victoria, Australia. For the purposes of this study, telehealth included consultations by both video and telephone calls. This study was part of a larger project involving a collaboration between universities, health care services, government agencies, and software developers, which sought to enhance an existing telehealth (video call) platform with additional features to improve both patient and HCP experience. By better understanding users’ experiences and perceptions of telehealth, we hoped to determine users’ preferences for other telehealth enhancement features.

## Methods

### Participants

Palliative care professionals and patients were recruited from the Monash Health Supportive and Palliative Care Unit at a tertiary hospital network in Victoria, Australia. HCPs, including palliative care medical and nursing staff, and interpreters involved in the Unit’s use of telehealth were invited to participate by email. The Unit has video telehealth palliative care clinics for oncology patients. In addition, during COVID-19 outbreak, the Unit provided a telehealth outreach service when in-person home visits were not possible.

Patients aged 18 years and older, with conversational English proficiency, currently using telehealth to access palliative care were invited through email invitations and postcards. A total of 6 patients and 6 HCPs participated in the study. All participants lived in Victoria and spoke English as their primary language. The patients’ ages ranged between 54 and 61 years old. In addition, 4 of the 6 patient participants identified as female and 2 identified as male. The HCPs’ ages ranged between 33 and 62 years old, with 5 identifying as female and 1 as male. The duration in their health care roles ranged from 6 months to 8 years. Their roles were ‘doctor’ (n=3), ‘interpreter’ (n=2), and ‘nursing clinical coordinator’ (n=1). Our 6 patient participants have been deidentified as P009, P012, P013, P014, P015, and P016, and HCP participants as HCP001, HCP003, HCP 004, HCP005, HCP010, and HCP011.

### Data Collection

The study used a qualitative photo interviewing technique [17,18]. Participants were requested to generate 3‐5 photographs based on certain prompts and share them with the research team. HCPs were asked to click photographs to depict their everyday work life in the context of telehealth models of care, what telehealth represents in their work, general experiences, and opportunities for improving current telehealth models. Similarly, patients were asked to generate photographs to illustrate their experience of receiving health care through telehealth, what telehealth represents for them as patients and their everyday lives, and opportunities for improving telehealth models of care. Multimedia Appendix 1 and Multimedia Appendix 2 shows the specific instructions provided to both HCPs and patients.

Participants emailed their photographs to a member of the research team. Subsequently, telephone interviews, lasting between 30 and 45 minutes, were conducted with each HCP and patient participant. During the interviews, participants were asked to elaborate on what each of the photos meant for them and how the photographs related to their experiences of telehealth. During the interviews, use of participant-generated photographs helped trigger participants’ memories and invoke their deeper consciousness, values, attitudes, and personal meanings of telehealth [19-21]. The visual aids also helped the researcher and participant reach a shared understanding of the topic [22]. All the interviews were audio-recorded and transcribed verbatim by a professional transcribing company. The interview transcripts were deidentified, and photos blurred to protect participants’ identities. The anonymized transcripts were imported into Microsoft Word and the photos inserted at the point where they were discussed during the interviews, allowing for an intertextual analysis in which the participants’ photos and words could be viewed together [23].

### Data Analysis

A multistep data analysis process was conducted, involving intertextual analysis wherein participants’ words and photos were viewed together and analyzed using a 3-step process involving (1) preview, (2) review, and (3) cross-photo comparison [23]. In the first “preview” stage, the first author reviewed all the photos and identified recurring motifs. These motifs were recorded in a PowerPoint slide deck and shared with the research team at a meeting for feedback and further interpretation. Subsequently, a more in-depth “review” was conducted wherein one researcher coded all the interview transcripts with relevant themes and subthemes in more granular detail. The emergent codebook was shared and workshopped with the research team to further distill the emerging patterns.

The final stage in the data analysis process was “cross-photo comparison.” During this phase, the research team discussed the similarities and differences within and across participant groups (eg, within patient participants’ or within HCPs’ accounts, or across patients’ and HCPs’ accounts). Often the patients’ and HCPs’ accounts provided contrasting experiences of the same facet of telehealth use (referred as “counter-examples”). For example, where patients valued the convenience of telehealth and being able to liaise with their doctors from anywhere, HCPs found patients’ use of telehealth in certain places (eg, public spaces) disruptive and uncongenial to the therapeutic process. To organize the emergent themes, a storyboard was developed by the first author containing illustrative quotes and photographs from both patients and HCPs. This story board has been provided in Multimedia Appendix 3 . The storyboard was presented to the research team and subsequently, manuscript development commenced.

### Ethical Considerations

Ethics approval for the study was obtained from the Monash Health Human Research Ethics Committee (project identification number 79681). All participants were provided plain language summaries of the research project, and what their participation would entail. All participants provided informed written consent. They also had the option to stop the interview at any point. Only approved members of the research team had access to the identifiable participant information. All interview transcripts were deidentified before the commencement of the data analysis process. Photos generated by participants showing any humans were also blurred to preserve participants’ privacy.

## Results

### Overview

A total of 3 themes were identified: comfort (or lack thereof) afforded by telehealth, connection considerations in telehealth, and care quality impacts of telehealth. It should be noted that while these themes are presented as distinct categories, they are not necessarily mutually exclusive. In addition, in some cases the themes included counter-examples, as mentioned above.

### Comfort (Or Lack Thereof) Afforded by Telehealth

For some participants, telehealth afforded them greater physical and psychological comfort. However, for some others, telehealth hampered their physical and psychological comfort. Therefore, this theme elucidates how telehealth use impacted patients’ and HCPs’ physical and psychological comfort.

### Physical Comfort

Palliative care patients often experience physical discomfort, including pain and fatigue, which in turn impacts their ability to visit clinics for in-person consultations. Patient participants advised that they had arranged their homes in ways that minimized their discomfort. Telehealth enabled them to remain in this environment, unlike in-person appointments, which lead to physical exertion due to travel and wait times, with this issue compounded for rural patients.

**Figure 1.**
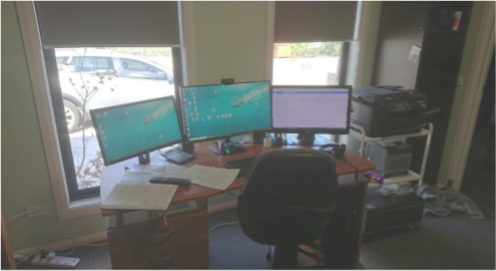
Patient home office for telehealth consultations (P015).

Patient participant P015 captured this theme when discussing a photo (Figure 1) of the room where they spent most of their time. They reported being “in pain 24/7” and explained how telehealth helped minimize their discomfort:

...You can actually see how close my bed; my chair is to my office desk. And so, I just use the walking frame, I get to the chair very easily. I sit in my chair, I’m listening to a bit of music, or I’m just watching TV…I’ll sit in the wait in the chair, waiting for the doctor to come in.

[P015]

**Figure 2.**
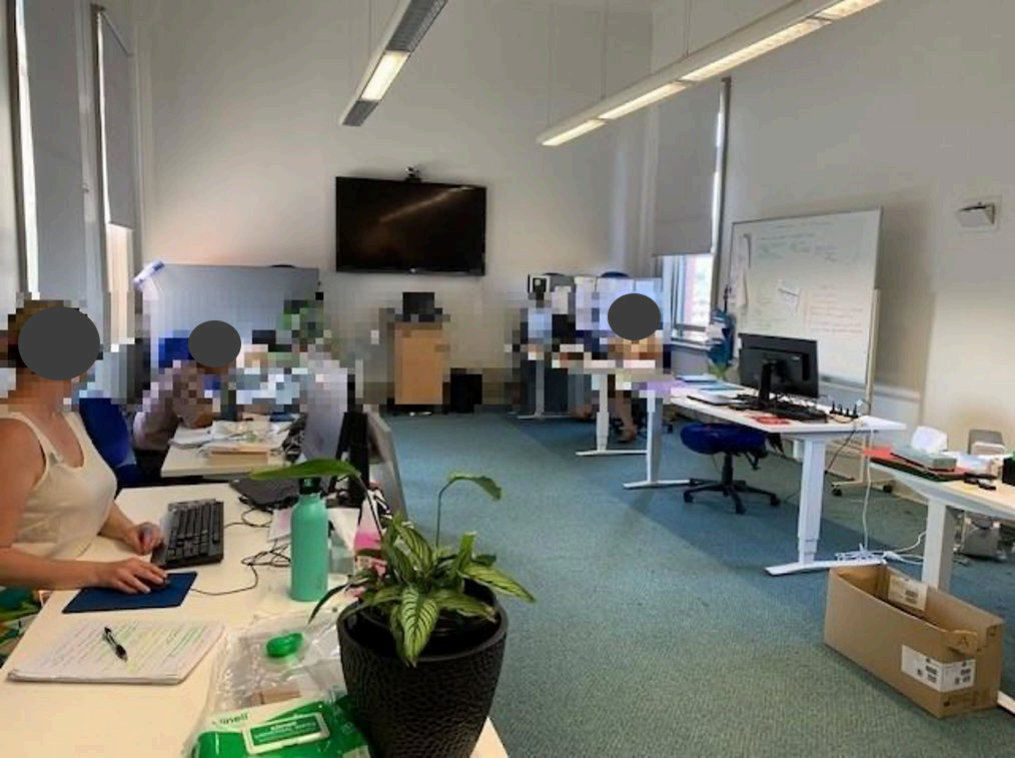
Palliative care service shared office space (HCP001).

By contrast, some HCPs associated telehealth with physical discomfort. HCPs working in the palliative care outreach service during the pandemic, as opposed to the Unit’s dedicated telehealth clinics, reported that private rooms were not always available. Therefore, telehealth appointments were often conducted in shared workspaces, which could be noisy and distracting, impacting their ability to effectively conduct quality consultations with patients. For example, HCP001 included a photo of the shared office space (Figure 2) where they often conducted telehealth consultations and stated:

It gets very loud because we’re [all] talking… So then anybody else talking on the phone, someone else has to talk up to talk over the top of that, and then anybody having a conversation with each other in the office has to talk up.

[HCP001]

### Psychological Comfort

**Figure 3.**
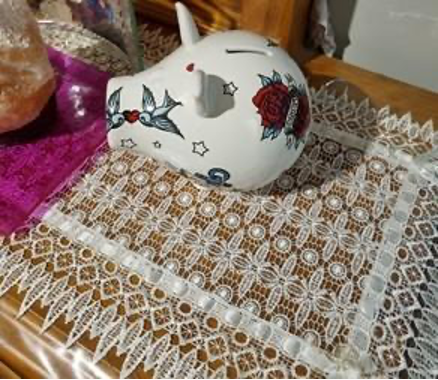
Piggy bank (P013).

Alongside physical comfort, telehealth provided patients with psychological comfort by reducing the stress associated with in-person appointments. Participants reported a variety of psychological stressors associated with in-person appointments, which were mitigated by using telehealth, for example, travel and wait times, risk of exposure to COVID-19 in hospitals, forgetting key questions for their doctors, and financial stress, particularly for rural patients who may need to make overnight accommodation arrangements for brief appointments. P013 provided a photo of a piggybank (Figure 3) and stated:

…going to appointments, car wear and tear, fuel for my daughter, parking fees, petrol... [With] telehealth, [I] don’t have to spend that money… [the money saved could be spent on] all the bits and bobs that they keep chucking at me to take.

[P013]

**Figure 4.**
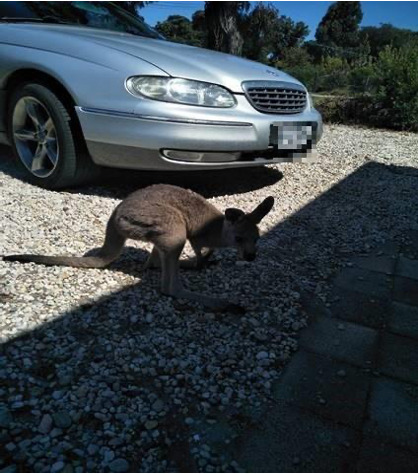
Life in ‘the bush’ (P014).

The psychological comfort associated with the freedom to stay in one’s chosen location and receive health care from the comfort of their own homes is also illustrated in P014’s photo (Figure 4) and quote as follows:

My bush family are my reason for living out here … This is my family. Because of telehealth, I get to stay home and enjoy them. You know, I don’t lose a day or two being on the road away from them. I know it’s only animals but for some people, you know, animals are our family.

[P014]

**Figure 5.**
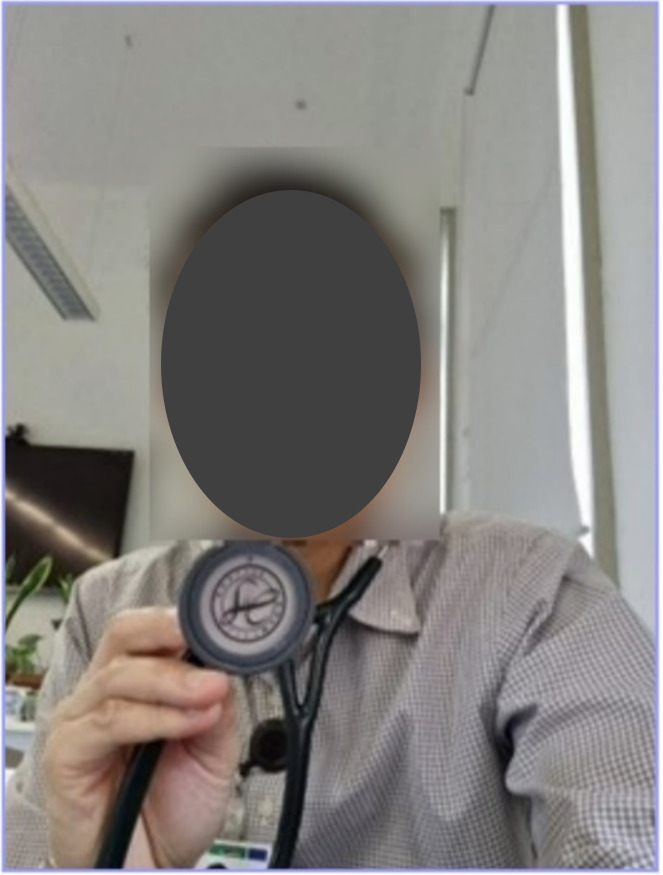
Health care professional (HCP) uncomfortable about limitations of telehealth (HCP005).

For some outreach service HCPs, however, the sudden large-scale transition to telehealth in the aftermath of the COVID-19 outbreak, led to some psychological discomfort at their perceived inability to provide their usual standard of care. Given their professional training had primarily been in face-to-face care, they felt ill-equipped to efficaciously transition to online care. HCP005 provided a photo of themselves holding their stethoscope up to the camera (Figure 5), demonstrating the limitation of physical examination in telehealth consultations. HCP005 said that the return to more in-person consultations was “almost a relief” post–COVID-19 pandemic. They further elaborated:

…often to be thorough in your assessment, as a doctor, you’ve been trained [to] do your history, examination and do investigations. I guess telehealth takes away … what we’re trained to do.

[HCP005]

### Connection Considerations in Telehealth

Participants indicated that 2 types of connection were imperative for effective telehealth delivery: technical connection (eg, good internet connectivity or clear phone line) and interpersonal connection (ie, good rapport and therapeutic alliance between HCPs and patients). Often technical connection issues impeded the development of interpersonal connection between the HCPs and patients in telehealth. This theme illustrates how technical connection and interpersonal connection impacted the participants’ telehealth experiences.

### Technical Connection

**Figure 6.**
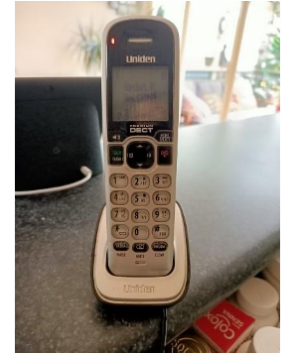
Landline home phone as back-up (P012).

The technical aspects of connection included the skills required to use the technology, internet and mobile phone connectivity, and specific hardware and software concerns. HCP participants noted that their patients had first been introduced to the telehealth model of care during the pandemic with varying levels of technological proficiency. Patients with limited technological proficiency needed to build their confidence and skills over time, with support from HCPs, support staff, and family members. HCPs described situations where no one had checked whether or not a patient’s computer had required microphone and video capabilities. Internet connectivity and bandwidth issues were also reported by both patients and HCPs. HCPs, in particular, due to their volume of telehealth calls, reported a lot of time lost to troubleshooting connectivity issues, which impacted the time available for clinical conversations. Often participants had to transition to telephone calls. For example, P012 shared an image of their landline home phone (Figure 6), describing it as follows:

It is the most reliable tool in my house. It never drops out. Whereas the Internet … the internet on my computer can be slow or drop out or whatever at a telehealth appointment. And my mobile phone drops out, it’s unreliable as well. But this phone is not unreliable. So if I lose a connection with any of the doctors, I know I can ring them on that phone … and it’s not going to drop out. That’s really important … So the landline is a good back up source for me for telehealth. I always have it sitting there in case something happens.

[P012]

**Figure 7.**
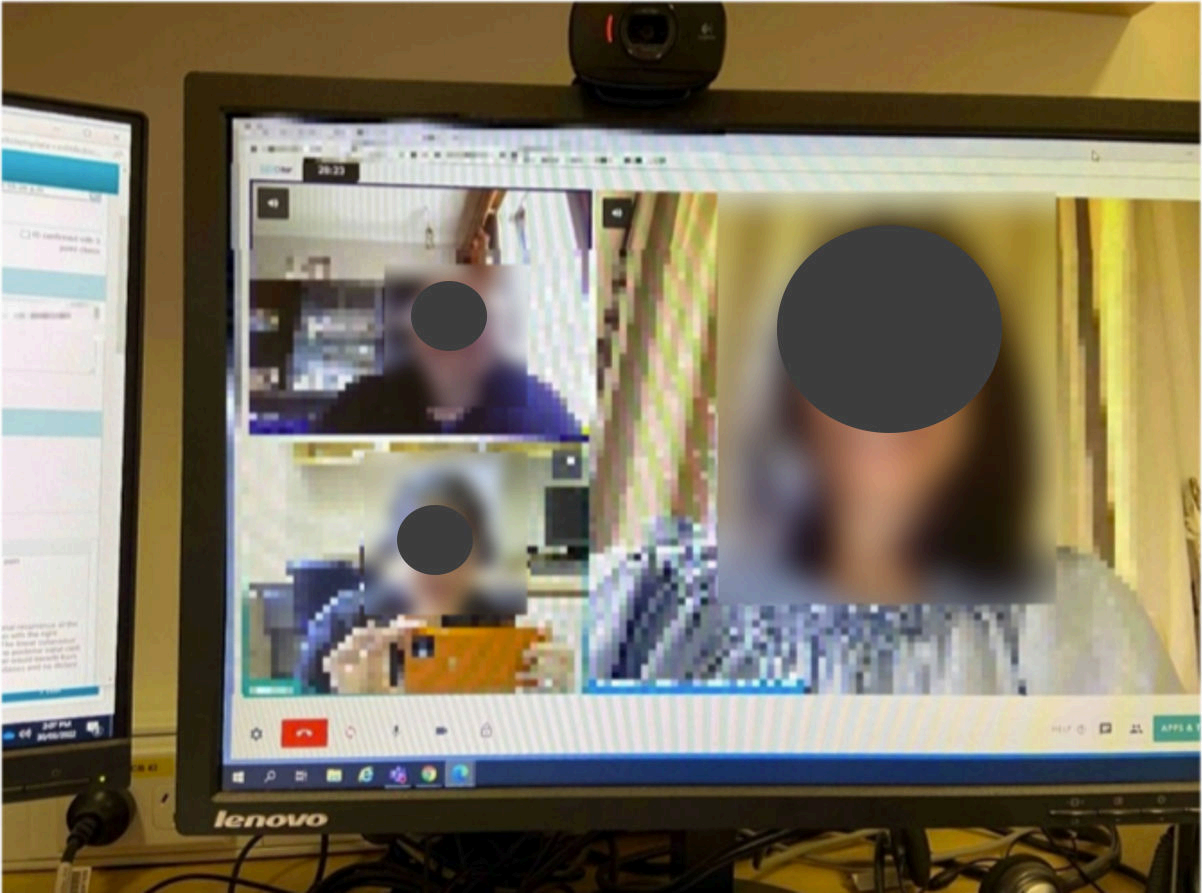
Positive experience of telehealth (HCP003).

Nevertheless, participants also reported that in the absence of connectivity issues, and with the appropriate skill level to use, and availability of the right technology, telehealth can be very useful and seamless. For example, HCP003 illustrated a good experience of using telehealth involving both a patient and their carer in Figure 7, and recounted:

… essentially showing how we can make it work when everyone is capable of using telehealth. On the right … that’s actually the patient’s son … But it just shows how it can work. So … having patient caregiver in telehealth, it is all very possible. The video worked well the audio worked well ... there was no barrier there.

[HCP003]

### Interpersonal Connection

**Figure 8.**
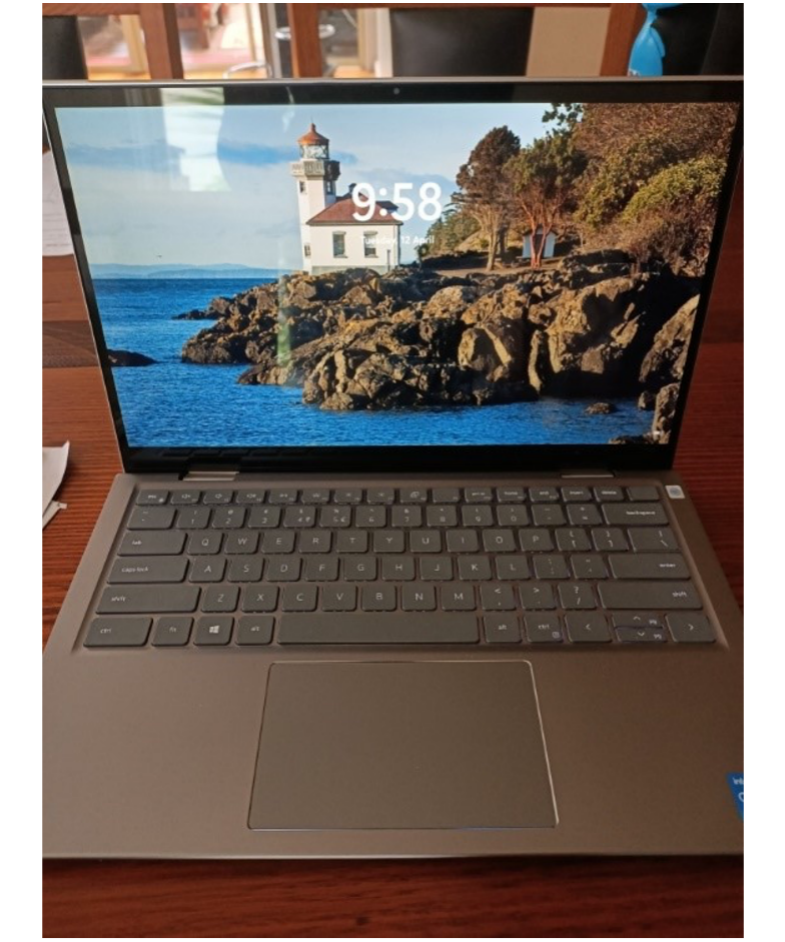
Patient’s laptop used for video consultations makes them feel more relaxed (P012).

Participants expressed a general preference for video consultations over telephone consultations because of more opportunities for eye contact, and reading of facial expressions or other nonverbal cues. The notion of power dynamics between HCPs and patients also emerged, with some participants stating that conducting the consultations over telehealth made patients feel more relaxed and open, as opposed to feeling nervous in front of their HCP in person. P012 provided an image of their laptop (Figure 8), which they used for video consultations and stated:

I’ve noticed for me that I open up and talk more ... But when I’m in his rooms, I’m intimidated when I’m in person with him, face to face. I’m more conscious of myself and self-conscious. Whereas over the computer I’m not. I’m really confident and I just pour everything out.

[P012]

**Figure 9.**
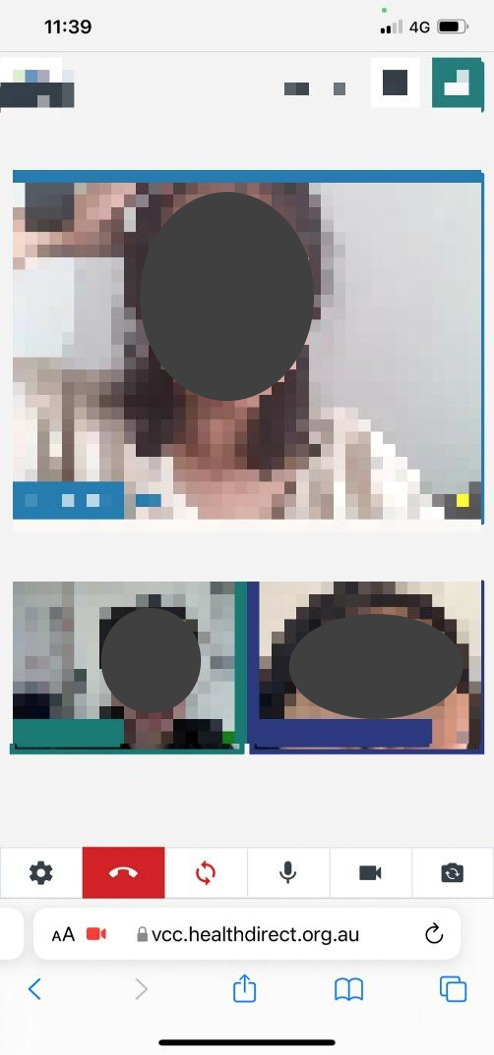
Video consultation conducted on a mobile phone (HCP011).

The type of technology used for video consultations also played a role in the quality of the clinical conversations. As previously mentioned, participants sometimes had to rely on backup options such as mobile phones or landlines for consultations due to technological failures, which also impacted the quality of their telehealth consultations. HCP011 shared a photo of an outreach service consultation that they had joined using their mobile phone, because the laptop and desktop were being used by other HCPs (Figure 9), and reflected:

The pictures are very small. So it just does not give you a realistic feeling of seeing a patient.

[HCP011]

In addition, participants generally agreed that periodic in-person consultations interspersed between their regular telehealth appointments were necessary to generate rapport between patients and HCPs. P015 had video telehealth oncology appointments every 6‐8 weeks for over a year, despite which they said, “I’ve never met my oncologist,” by which they meant that they had never met their oncologist in person. Even P012, who felt they were able to be more open through telehealth said:

the doctors should touch base with you specifically in person after a period of at least six months…. seeing them person to person sort of reconnects the relationship.

[P012]

In considering the benefits of in-person care, HCP010 said:

…we still want that relationship with the patient, we want to look them in the face and an important aspect of interpreting is body language. So even if you see them on the screen, [it] is not the same.

[HCP010]

HCP005 explained that some patients, “feel a bit suspicious or not completely trusting when you are not there [in person].”

Separately, HCP004 and HCP005 both also said that breaking bad news was more difficult through telehealth because of their inability to offer comfort through nonverbal communication like touch.

### Care Quality Impacts of Telehealth

This final theme elucidates participants’ perceptions about how telehealth impacts the quality of health care for patients.

Patient participants expressed mixed feelings regarding their satisfaction with the telehealth model of health care. While some participants perceived that the care they received through telehealth was satisfactory, others expressed concerns around issues such as lack of physical examination, impact on treatment timeliness, and communication.

Patients who described telehealth care positively viewed the crucial elements of care as unaffected. P014 explained that being able to share documents and show the HCP medications meant that:

[telehealth] doesn’t stop the communication process in anyway…as [a] patient I’m not missing anything.

[P014]

Participant P014 was also able to monitor their blood pressure and oxygen saturation at home and share this information with their HCP. HCP004 saw telehealth as working well when there was another complementary team, for example, “hospital-in-the-home,” which offered in-person care and provided clinical information to the palliative care patients. They also described the benefits of using telehealth to determine when an in-person visit was necessary:

it works best where there’s opportunity for, or being able to identify that you can’t rely on telehealth alone.

[HCP004]

**Figure 10.**
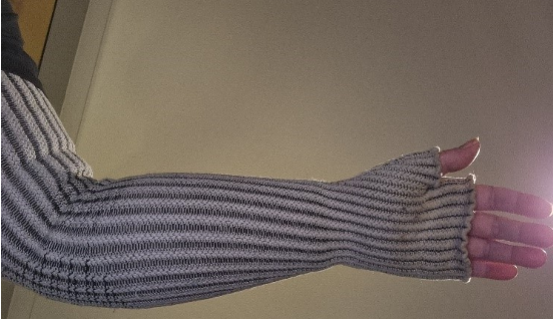
Lymphoedema sleeve (P013).

Participants who believed that the lack of an in-person examination had compromised their care were more negative about telehealth. For example, P013 shared a photo of their arm in their lymphoedema sleeve (Figure 10) to describe the way telehealth had limited their care:

I was seeing her [my HCP] pretty much monthly on telehealth. As my arm would flare up, there was still not much she could do because we weren’t allowed in … she finally got face to face with me, measured me up for the sleeve, so that was six months without proper diagnosis.

[P013]

This participant perceived that their symptoms had not been taken as seriously or treated as effectively because the HCPs involved had not been able to examine them.

**Figure 11.**
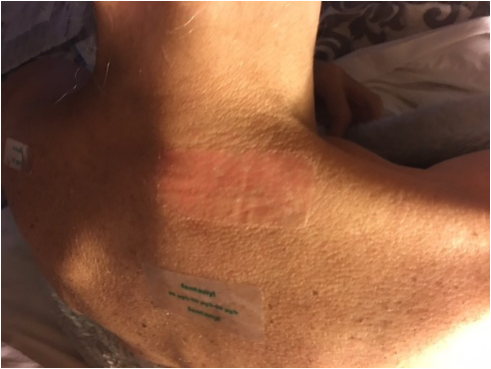
Patient finding it difficult to show the health care professional (HCP) her skin redness concern (P016).

P016 used a photo of their skin (Figure 11) to discuss the implications of limited physical examination opportunities in telehealth:

I was trying to explain to the doctor what the problem was … he actually couldn’t see me, what I was trying to explain he couldn’t see … And we didn’t really get to the bottom of what it was.

[P016]

For this participant, in-person care was important not only in this specific instance, but also because:

the doctor would pick up from the last time he saw you or your oncologist who last saw you [on] sort of things that you don’t notice in yourself … I just feel as I’m getting more unwell, that I need to have more face-to-face appointments than telehealth appointments, but they tend to push you towards telehealth.

[P016]

HCPs also stated that telehealth sometimes resulted in less thorough assessments. HCP001 explained that this may be the result of poor technical connection, whereas HCP005 described it as a problem when the assessment required a third party to facilitate the telehealth consultation, such as staff in an aged care facility, whose other duties meant they did not have the requisite time to devote to the consultation.

**Figure 12.**
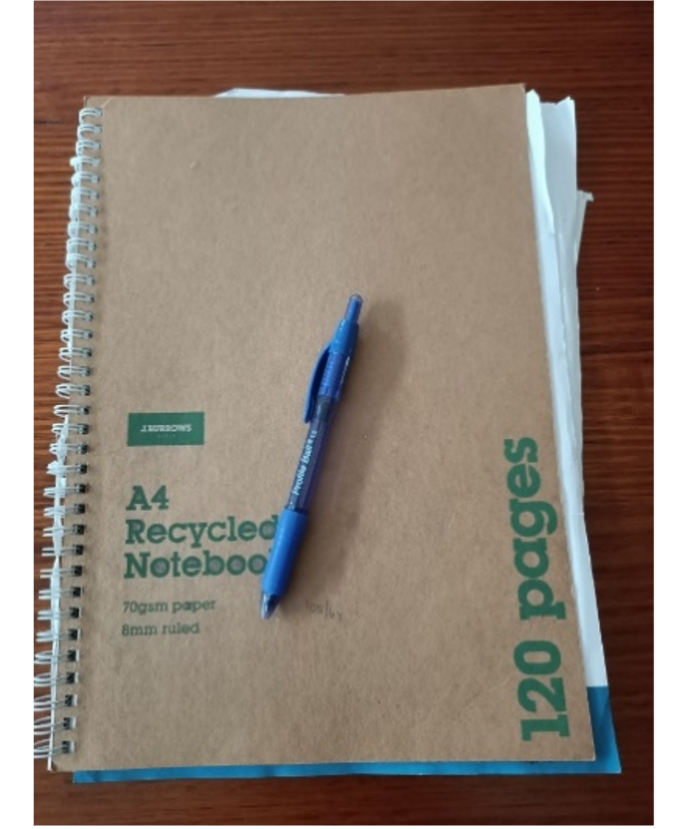
Patient feels more in control of their ability to take notes before and during telehealth consultations (P012).

On the other hand, a positive aspect of the telehealth model of care for patients was their ability to feel more in control of their self-care and self-management during telehealth consultations. For example, patient P012 took a photo of their notebook (Figure 12) to illustrate that they felt more comfortable bringing prewritten notes for discussion and scribing notes during a telehealth consultation than they would in person:

Notebook [is] very important ... Leading up to all my appointments I have, I make notes. And then when my appointment comes up, I cross stuff off … that’s really important. For me, it’s my notes. That way, I know I’m not going to forget anything. And whatever they say to me that’s relevant or important, something I had to do or whatever, I’ll write it down … I probably wouldn’t do that at the doctor’s … But online I can just do it on the side while I’m talking to them.

[P012]

Similarly, another patient P009 recounted a similar experience of feeling more comfortable about their ability to capture important information before and during a telehealth consultation by having family members present with them from the comfort of their homes:

Part of the brain tumors is that I can’t remember stuff afterwards. So I have to have someone there who’s like my virtual notepad … they can answer things that I may have forgotten especially to do with medication because it seems to be the main one or [one of the] side effects … So it works well as in, they’ve [husband and/or daughter] got an area where they can be comfortable … I have a pad and pen here. But of course I have sometimes husband and/or daughter not only remembering stuff like that’s said but going back and reminding me of stuff I need to tell the oncologist that I may have forgotten about.

[P009]

## Discussion

### Principal Findings

Our intertextual analysis yielded 3 key themes: comfort (or lack thereof) afforded by telehealth, connection considerations in telehealth, and care quality impacts of telehealth. Participants’ relationship to telehealth was nuanced. Generally, participants acknowledged the myriad of benefits that telehealth brings, such as greater psychological and physical comfort for patients, especially those based in rural areas. However, there was also a general sense that telehealth should be combined with in-person care for optimal patient-HCP therapeutic alliance and better patient outcomes. It should be noted that this study’s data collection commenced during the pandemic, amid nation-wide efforts to rapidly scale telehealth models of care across Australia. Eastman et al [15] sought to understand patients’ and HCPs’ experiences of telehealth in community palliative care. Their findings were congruent with ours, suggesting that telehealth is being increasingly used in palliative care and generally patients and HCPs are supportive of using this model of health care, combined with regular in-person touchpoints.

A salient finding from our study was the notion of greater power symmetry between patients and HCPs in telehealth compared with in-person consultations. Patients alluded to feeling more empowered, in control, and psychologically comfortable during teleconsultations. This translated into them feeling more confident about “pouring out” information in front of their HCPs, than they would face-to-face. Furthermore, the safety of a device’s screen afforded patients the space to take notes during consultations. Having teleconsultations from the comfort of their homes where family members could also be present where needed, meant that patients felt more at ease. In some cases, family members also served the important function of remembering what had been discussed during consultations, which enhanced patients’ quality of care.

Our team conducted additional co-design activities with consumers which are outside the scope of this article. In those activities too, participants had reported the importance of taking notes before, during, and after their telehealth appointments. Participants described forgetting important details from their appointments and relying on their family to attend consultations to remember what had happened during the appointment.

Literature suggests that 40%‐80% of medical information and recommendations provided by HCPs during clinical consultations is forgotten by patients almost immediately. The poor information recall may have downstream impacts for treatment adherence [24]. Issues of memory recall may be exacerbated in palliative care patients who experience complex illness symptomatology. There is some evidence to suggest that patient-facing clinical consultation recordings can be helpful for patients in general. A scoping review conducted by Tsulukidze et al [25] in 2014 suggested that patients place a high value on audio recordings of clinical consultations and benefit from subsequently listening to the consultation recordings.

The participants of our study reported in this paper and subsequent co-design activities indicated that having a summary of the consultation at the end of telehealth session would mean that this information is kept secure and in one place for patients. Memory and cognitive deficits are often experienced by palliative care patients. Therefore, a mechanism to record salient information discussed during consultations would be particularly useful for this patient population.

The literature on patient-facing consultation summaries generated during telehealth is currently limited. A non-systematic literature scan in Google Scholar on this subject yielded no relevant papers. The closest relevant literature appears to be about artificial intelligence (AI) enabled digital scribes or documentation systems, which can help automate clinical documentation tasks ordinarily conducted by humans [26-28]. However, the literature on patient-facing clinical summaries in telehealth (whether generated manually by HCPs or through AI-enabled algorithms) is not yet developed.

The paucity of literature on this subject combined with the participants’ unanimous views emergent in our investigations, emphasized the need for innovating in the area of patient-facing clinical consultation summaries. Since the conduct of the study presented in this paper, our team of software developers has created a telehealth-enhancement add-on feature which seeks to generate patient-facing summaries of clinical consultations during video telehealth sessions. The prototype was developed in an agile manner, involving think-aloud testing activities with end users. Subsequently, the minimum viable product (MVP) was subjected to a simulation study to generate early-stage evidence about end-users’ perceptions of the add-on’s potential clinical usefulness. The findings of our think-aloud and simulation studies will be reported in future papers. However, based on participants’ accounts presented in this paper, we expect that the provision of a patient-facing document that summarizes the telehealth consultation and distills key actionable clinical advice for patient self-management, might enhance patient experience and self-management.

### Limitations

The limitations of this project include the small number of participants interviewed. The small sample size was partly due to challenges in recruiting palliative care patients. Patients’ complex symptomatology and in some cases, limited life expectancy, impacts their ability to participate in research studies. In addition, as the data collection was conducted during the pandemic, this posed additional challenges for HCP recruitment. Nevertheless, our multimodal data collection approach involving the use of photographs and interviews combined with dual perspectives of patients and HCPs meant that we had a rich dataset to generate an in-depth understanding of users’ experiences of telehealth.

In this instance, we did not collect information about, or pose eligibility restrictions regarding participants’ ethnic and racial background, socioeconomic status, and level of experience with telehealth. This decision was driven by the small number of palliative care participant population available to participate in the study. Future telehealth research may include contextualisation of emergent findings through the lens of one or more of these socio-demographic characteristics.

### Conclusion

This study explored patients’ and HCPs’ experiences of telehealth in a palliative care context. A total of 3 themes emerged: comfort (or lack thereof) afforded by telehealth, connection considerations in telehealth, and care quality impacts of telehealth. The findings presented in this article combined with other co-design activities, which are outside the scope of this paper, indicated the potential value of a telehealth enhancement add-on feature that generates patient-facing clinical consultation summaries. Our team has developed a video telehealth enhancement feature, which will enable clinicians to distill key actionable advice and self-management guidance discussed during teleconsultations in a take-home summary document for patients. The add-on prototype has been subjected to a preliminary simulation study which will be reported in a future publication.

## Supplementary material

10.2196/53913 Multimedia Appendix 1 Photo instructions—health care professionals (HCPs).

10.2196/53913 Multimedia Appendix 2 Photo instructions—patients.

10.2196/53913 Multimedia Appendix 3 The 3 Cs storyboard.

## Abbreviations

AI: artificial intelligence
HCP: health care professional
MVP: minimum viable product
