# Supplementary material for: Understanding Experiences of Telehealth in Palliative Care: Photo Interview Study
Source: JMIR Hum Factors. 2025 Feb 11;12:e53913. doi: 10.2196/53913 (PMC11835783; doi:10.2196/53913)
Supplement: Multimedia Appendix 2 [file humanfactors-v12-e53913-s002.pdf]

Your experiences in pictures – for patients & caregivers

Thank you for being a part of this project focused on Enhanced Telehealth Capabilities (ETC) in palliative care and mental health settings and for agreeing to be interviewed about your experiences with current telehealth models as a patient. In preparation for our interview, we have asked you to create a few digital photos that help when we talk using phone or internet. The photos should tell us about:

- YOU !
- We are interested in you taking three to five photos before your interview that help to tell us about your everyday care – what do you do for telehealth appointments?
- We would like you to think about what telehealth represents to you as a patient, share your experiences and take a photo or two that represents how it could be better.

What should the photos be about?

Over the next week or two leading up to our telephone interview, we want you to feel free to take photos using your smart phone.

We ask you take at least three to five photos you are prepared to share and use within the interview, but please do not send more than five due to the time available for the interview.

Once you've taken the photos we ask you to email them ahead to the research team at pcmh-gp@unimelb.edu.au who will have these at hand for the interview. We ask you to send the email with the photos attached, and explain in one or two sentences what prompted the photo and why it is important to the interview for you and the topic of enhanced telehealth care.

You might want to take photos when you are on your own or when you are with other people – if the photo has people in your family in it we will need their consent to be seen by other people. Your photos might include meaningful people, places, activities or items from your care and receiving telehealth care. The photos can be of ordinary things or unique parts of being a patient with telehealth care that are important to you.

The tips at the end of this sheet might be helpful. The points listed below are some general things you might like to take photos of you to explore further in the interview; you don't need to include these photos if you don't want to:

- Places that are important to you in telehealth care delivery
- A challenging time in telehealth care
- How you feel the models of telehealth could be enhanced

Sharing the Photos

When you are ready to share your photos, contact the research team by email with them. ***Please remember to include a few sentences about what the photo relates to and why you took it*** – does it relate to a difficult day or encounter using telehealth? Or, an activity you have to do related to telehealth? Or, is it about something that could be better in telehealth?

When you take part in your interview, the researcher will ask you to select the photo you want to start with and for you to explain why that photo is important to you for enhanced telehealth capabilities—there will be some guiding questions to help with this.

For this study, you will be talking with a researcher from the Centre for Digital Transformation of Health and researchers from the ALIVE national centre for mental health research translation at the University of Melbourne. We'll let you know who they are before the interview and check if you need anything specific to link up with them. During the conversation you and the researcher can look at the photos together using skype or zoom, phone is okay too if both the interviewer and interviewee can see the same photo.

The photos are designed to facilitate the interview conversation, and these may also be helpful to use in future workshops about co-designing enhanced telehealth capabilities in the palliative care or mental health settings; we will check back with you to make sure you are okay with sharing any particular photo and the explanation that goes with it. The photos may also be used to create a digital story about patient experiences of telehealth during the COVID-19 pandemic.

Tips for Taking Digital Photos

These tips are just to help you get started with your photos.

Thinking About Light

- Photographs only exist because there is light, so it is a great idea to consider what sort of light you are taking your photos in.
- It often works best if the light is coming from behind the photographer. That's likely to be you most of the time - but it is OK if you want to get someone else to take a photo of you at some point to help create your visual story—please do not take photos with any patients in them if you are taking these at work; it is okay to take photos of other staff who agree and give written permission after for their photos to be used.
- The more light the better! Outdoors during the day is a great time to take shots, but indoors can work too.
- If there isn't a lot of light indoors so you may want to use some household lamps if you have some.

Trying Something Different

- Have a go at taking some shots at different angles.
- You might want to take a series of photos of one object from several different views. For example, one from above, one from below or one from the side.
- Try moving closer to an object, or further away from it.
- You may want to take a picture of an object right up close because that's all you want the photo to be about.

Asking Permission

- It’s always a good idea to make sure that the people you photograph don’t mind being in your photos.
- Please ask people before photographing them—and if there is someone in the photo and it is not you we will need them to have signed a consent form that we gave you just in case for this too.

GENERAL TIPS

Try not to think TOO much!

- There are no right or wrong photos for this project.
(Photos you provide will be confidential 'subject to legal requirements' – this will be explained to you further by the researcher)

- We’re interested in whatever you want to show us.

- Be spontaneous and take ordinary photos if you want to.

- If you feel like being more creative - don’t be afraid to try different things, if something doesn’t work out, don’t worry, that’s photography!

- This is your opportunity to share visually how telehealth currently happens for you and ways you would represent visually how it could be better – enjoy!
