# Supplementary material for: Understanding Experiences of Telehealth in Palliative Care: Photo Interview Study
Source: JMIR Hum Factors. 2025 Feb 11;12:e53913. doi: 10.2196/53913 (PMC11835783; doi:10.2196/53913)
Supplement: Multimedia Appendix 3 [file humanfactors-v12-e53913-s003.pdf]

Patients

COMFORT

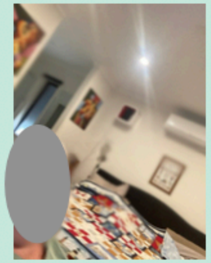

Picture 009-3

"I'm relaxed and comfortable... So it's fairly smoothly like I know I can come in here. It takes me about five minutes to set up and then I'm all ready to go and because you're looking at somewhere where you sort of got to be for 45 minutes to an hour sometimes longer... Everything I need like I have a pad and pen here ... I [also] have sometimes husband and or daughter not only remembering stuff... but going back and reminding me of stuff I need to tell the oncologist that I may have forgotten about."

Patients feel comfortable when attending consultations from their home environment and appreciate the ability to include family members in these conversations.

CONNECTION

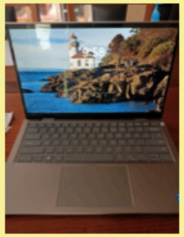

Picture 012-5

"When I'm over the phone, if I have an appointment, I'm talking million miles an hour... I feel like I don't want to waste their time, like get off the phone as quickly as possible... But over the computer I'm not like this. On the phone I put pressure on myself to rush the appointment. Things I've forgotten to talk about even though I might use a list, we get sidetracked. And then and then I think 'How long have you been on the phone?' I can't see how long I've been talking to the doctor for and I'm very conscious. And I'm probably on the phone for about three to five minutes... [But on video], you can relax and you can talk to them. You can see their facial expressions. They may talk slower and more relaxed and it makes you slower and the conversations better than over the phone... You can see them sitting there... And I reckon it should be that the doctors should touch base with you specifically in person after a period at least [every] six months... Seeing them person to person sort of reconnects the relationship... You're not just a visual person over the computer... I think once every six months, would be a good thing might be a requirement that the doctor should see you if possible."

Patients find it easier to form a therapeutic connection with their healthcare providers through video calls, as compared to telephone (or audio) consultations. They also expressed the need for periodic in-person check-ins to strengthen the therapeutic relationship with their doctors.

CARE

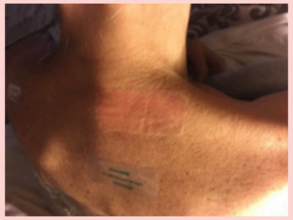

Picture 016-1

"I was trying to explain to the doctor what the problem was... What I was trying to explain, he couldn't see it. He said, it's hard to tell whether it's having an allergic reaction or whether it was just the redness from pulling off the patch... it was just really hard for them to determine what it was ... because they couldn't see me face to face... And we really didn't get to the bottom of what it was. And they just sort of said... just monitor it... And the other thing is I'm not going to see the doctor face to face, so they can't take blood pressure or listen to your heart or you know, and see see changes in you that that you wouldn't notice yourself."

The limited opportunities for physical examination via Telehealth was reported to be a source of frustration (by both patients and professionals). Patients also felt apprehensive about other health issues which may not be identified in a timely manner without in-person examination.

Healthcare Professionals

COMFORT

Healthcare professionals can find the clinical Telehealth environment uncomfortable and disruptive to the therapeutic process. Special technical and physical infrastructure is needed to make Telehealth comfortable and effective for professionals.

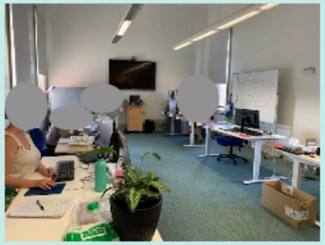

Picture 001-3

"It gets very loud because we're talking maybe in a telehealth, if it was [name removed for confidentiality] and myself, so nursing and medical, we create noise. So then anybody else talking on the phone, someone else has to talk up to talk over the top of that, and then anybody having a conversation with each other in the office has to talk up. So it can be it can be a barrier to having a large open plan sort of space, whereas I've heard of things like that, like telehealth, almost pods that have good audio acoustics within the pod, but then you can't hear much outside of that that position or sitting in that pod. So I could ... see the benefit or the value longer term in having a specific environment I guess for clinical work."

CONNECTION

Healthcare professionals often found Telehealth challenging from both patient connection and peer connection perspectives. They reported feeling isolated from both their patients and colleagues.

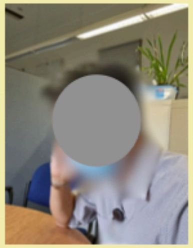

Picture 005-3

"It's just such a different model of medicine. Like... I went personally almost 12 months without seeing a patient in person. And yeah, it was just a huge sort of readjustment. And then finally, being able to see a patient ... it was it was almost a relief... Our unit certainly had a lot of staff on and off with COVID or being isolated. So certainly some of our work was being done from home at some point. And I think that was also quite a different experience ... it can be quite isolating... As a team, you know, there'd be a lot of chat in the office about sort of the work ... it's just missing... when you're not sort of in the same room... you definitely missed out on that."

CARE

Healthcare professionals reported that delivering appropriate care via video consults requires special observational skills. These skills can be strengthened with suitable training and experience in identification of visual cues.

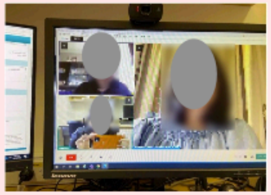

Picture 003-2

"... The video actually gives a lot of information when ... your consult is with someone who's lying in bed, as opposed to someone who's fully dressed and sitting up ... at the dining table. So there's a lot that the video can give... Telehealth itself is a very different sort of model of care in terms of the communication that we need to provide to patients versus face to face. So I think I would say over time... we kind of just develop our own methods and skill sets... But it's definitely we rely on many different cues through a video consult versus an audio consult phone alone versus face to face. I don't think there's training to, or what I'm not aware of any sort of education or any sort of workshops to really highlight the differences and how clinicians can actually be better."
